# Delayed Cerebral Ischemia as Progressive Neurovascular Unit Failure: A Hypothesis-Generating Perspective

**DOI:** 10.3390/ijms272110048

**Published:** 2026-11-09

**Authors:** Kareem Loubani, Ayesha Deed, Zubia Hafeez, Maleeha Urooj Farhan, Thaabit Raziq, Zahra Shafeeq, Saniyah Shaikh, Olena Bolgova, Ahmed Yaqinuddin, Volodymyr Mavrych

**Affiliations:** College of Medicine, Alfaisal University, Riyadh 11533, Saudi Arabia; kloubani@alfaisal.edu (K.L.); adeed@alfaisal.edu (A.D.); zhafeez@alfaisal.edu (Z.H.); mfarhan@alfaisal.edu (M.U.F.); mraziq@alfaisal.edu (T.R.); zshafeeq@alfaisal.edu (Z.S.); sanshaikh@alfaisal.edu (S.S.); obolgova@alfaisal.edu (O.B.); ayaqinuddin@alfaisal.edu (A.Y.)

**Keywords:** delayed cerebral ischemia, subarachnoid hemorrhage, neurovascular unit, cerebral vasospasm, endothelial dysfunction, microcirculatory dysfunction, neuroinflammation

## Abstract

Despite decades of investigation, delayed cerebral ischemia (DCI) remains the principal cause of secondary neurological injury following aneurysmal subarachnoid hemorrhage (aSAH). Successive paradigms including angiographic vasospasm, early brain injury, neuroinflammation, blood–brain barrier disruption, cortical spreading depolarization, microvascular dysfunction, oxidative stress, and metabolic failure have each explained important aspects of disease biology. However, these mechanisms have largely evolved as parallel explanations rather than components of an integrated model, leaving the dissociation between vasospasm and DCI, delayed onset, interpatient heterogeneity, and limited efficacy of vasospasm-directed therapies incompletely explained. This perspective proposes, as a hypothesis, progressive neurovascular unit (NVU) failure as a conceptual framework for DCI after aSAH. We integrate experimental, translational, and clinical evidence spanning vascular biology, neuroimmunology, cerebrovascular physiology, electrophysiology, metabolism, biomarker research, and therapeutic trials to develop a hypothesis-generating framework. The established mechanisms of DCI may represent interacting manifestations of progressive disruption of coordinated signaling among endothelial, vascular smooth muscle, pericyte, astrocytic, microglial, and neuronal compartments of the neurovascular unit. Within this framework, early brain injury may initiate NVU dysfunction, which is amplified by interacting vascular, barrier, inflammatory, electrophysiological, and metabolic disturbances, culminating in neuronal metabolic failure and tissue injury. Vasospasm is therefore positioned as one contributor to this process rather than a sufficient explanation for DCI. This framework offers a possible basis for interpreting clinical heterogeneity and therapeutic discordance while generating testable predictions for multimodal biomarker development, biological phenotyping, and mechanism-informed therapeutic strategies. The framework does not introduce new mechanisms; rather, it proposes a testable distinction between compensated NVU dysfunction and NVU failure, frames compartment-specific non-additive interactions as an empirical question, and organizes biomarkers by the NVU compartment they reflect. These proposals remain to be validated.

## 1. Introduction

### 1.1. Evolution of DCI Concepts

For several decades, delayed cerebral ischemia (DCI) following aneurysmal subarachnoid hemorrhage (aSAH) was viewed primarily as a consequence of angiographic vasospasm. Delayed narrowing of the large cerebral arteries was considered the principal cause of cerebral hypoperfusion, infarction, and subsequent neurological deterioration, making vasospasm the primary therapeutic target [1,2].

This paradigm was challenged by the CONSCIOUS-1 trial, which demonstrated that clazosentan significantly reduced angiographic vasospasm without improving functional outcome [3]. Subsequent reviews confirmed that therapies targeting vasospasm alone did not consistently improve disability or mortality, whereas reductions in delayed cerebral infarction were more closely associated with neurological recovery [1,4]. These findings suggested that vasospasm is an important manifestation of secondary injury but not its sole determinant.

Attention shifted to the earliest phase after hemorrhage. Sehba, Pluta, and Zhang introduced the concept of early brain injury (EBI), describing the immediate pathological response to aneurysm rupture during the first 72 h, characterized by transient global ischemia, oxidative stress, neuroinflammation, metabolic dysregulation, apoptosis, and blood–brain barrier disruption [5]. Rather than resolving after the acute phase, these disturbances initiate processes that predispose the brain to delayed neurological deterioration.

At the same time, growing evidence also demonstrates disturbances across microcirculatory regulation, metabolic homeostasis, neuroimmune signaling, and cerebrovascular control, indicating that delayed injury reflects dysfunction of an integrated neurovascular network rather than isolated pathological processes [6,7]. Although each mechanism is supported by substantial evidence, they have largely been studied independently. The field has therefore progressed from a single-mechanism explanation to multiple validated contributors, but how these processes interact to produce delayed cerebral ischemia remains incompletely understood.

### 1.2. Clinical Observations That Existing Models Cannot Fully Explain

If DCI resulted predominantly from a single pathological mechanism, its clinical manifestations would be expected to follow predictable relationships with that mechanism. Instead, several consistent clinical observations challenge this assumption and suggest that delayed neurological deterioration arises from progressive disruption of an integrated neurovascular system.

The clearest example is the dissociation between angiographic vasospasm and clinical outcome. Although approximately 70% of patients develop angiographic vasospasm, only about 30% develop clinical DCI [1,5]. Similarly, the CONSCIOUS trials demonstrated that reducing vasospasm did not improve functional recovery [3,4]. Conversely, delayed cerebral infarction may occur without significant angiographic vasospasm or in territories unrelated to arterial narrowing [1,2]. Together, these findings indicate that large-vessel vasospasm contributes to DCI but cannot independently explain its development.

Additional observations support this conclusion. Endovascular rescue therapies frequently restore arterial caliber without consistently improving neurological outcome, whereas nimodipine improves outcome despite having relatively little effect on angiographic vasospasm [4,5]. Furthermore, cerebral microdialysis detects metabolic crisis before perfusion abnormalities become evident on imaging, while microvascular dysfunction may persist despite angiographically normal proximal vessels [6,7]. These findings suggest that tissue-level dysfunction and impaired neurovascular regulation may evolve independently of large-vessel abnormalities.

Marked interpatient variability provides further evidence against a single-mechanism model. Patients with similar hemorrhage severity often demonstrate markedly different autoregulatory capacity, metabolic responses, and neurological outcomes. Measures such as the pressure reactivity index and lactate-to-pyruvate ratio independently predict progression to DCI-related infarction, while DCI-related deterioration may remain clinically undetected in poor-grade patients [6,7]. Therefore, these observations suggest the value of a conceptual framework that integrates, rather than replaces, the recognized mechanisms of DCI.

### 1.3. From Multiple Mechanisms to Testable Interactions

Recognition that delayed cerebral ischemia is multifactorial represents important progress, but it does not, by itself, constitute a disease model. Current evidence supports the contribution of vasospasm, microvascular dysfunction, neuroinflammation, cortical spreading depolarizations, blood–brain barrier disruption, metabolic dysfunction, and impaired autoregulation to secondary brain injury after aneurysmal subarachnoid hemorrhage [4,6,7,8,9]. However, identifying multiple contributing mechanisms does not by itself specify how they interact, which processes initiate disease, or how they collectively produce delayed neurological deterioration. Existing multifactorial accounts already recognize that these mechanisms interact and may act synergistically [4,6]; the framework proposed here does not claim otherwise but attempts to organize these interactions around the NVU and to state them in testable form.

An effective disease model should identify the biological system undergoing progressive dysfunction, distinguish initiating events from downstream consequences, and explain how interacting mechanisms amplify injury over time. Rather than viewing established mechanisms as parallel or competing processes, a systems-based framework should explain how they converge to produce the clinical syndrome of DCI [4,6].

The central challenge is therefore no longer determining whether these mechanisms contribute to DCI, as the evidence indicates that they do [4,6,7,8,9]. Instead, the unresolved question is how these validated processes are integrated into a common biological framework that explains the temporal evolution, clinical heterogeneity, and therapeutic response of delayed cerebral ischemia. Addressing this question provides the rationale for proposing progressive neurovascular unit failure as the hypothesis-generating framework explored in this review.

### 1.4. Central Hypothesis

The purpose of this perspective is to propose, as a hypothesis, a systems-based framework in which progressive neurovascular unit (NVU) failure serves as an organizing concept for delayed cerebral ischemia (DCI) following aneurysmal subarachnoid hemorrhage.

The neurovascular unit is the functional biological system responsible for maintaining cerebral homeostasis through continuous reciprocal communication among vascular, glial, immune, and neuronal compartments. Through this coordinated network, the NVU regulates cerebral perfusion, blood–brain barrier integrity, metabolic homeostasis, immune signaling, and neuronal function. Early brain injury is thought to initiate disruption of this network, which may produce progressive neurovascular dysfunction that is subsequently amplified through microcirculatory impairment, blood–brain barrier disruption, neuroinflammatory signaling, and metabolic dysregulation [5,6,7,8,9].

Accordingly, we define neurovascular unit failure as the progressive loss of coordinated communication among the vascular, glial, immune, and neuronal compartments, resulting in the inability to maintain cerebral perfusion, metabolic homeostasis, and blood–brain barrier integrity.

**Figure 1:**
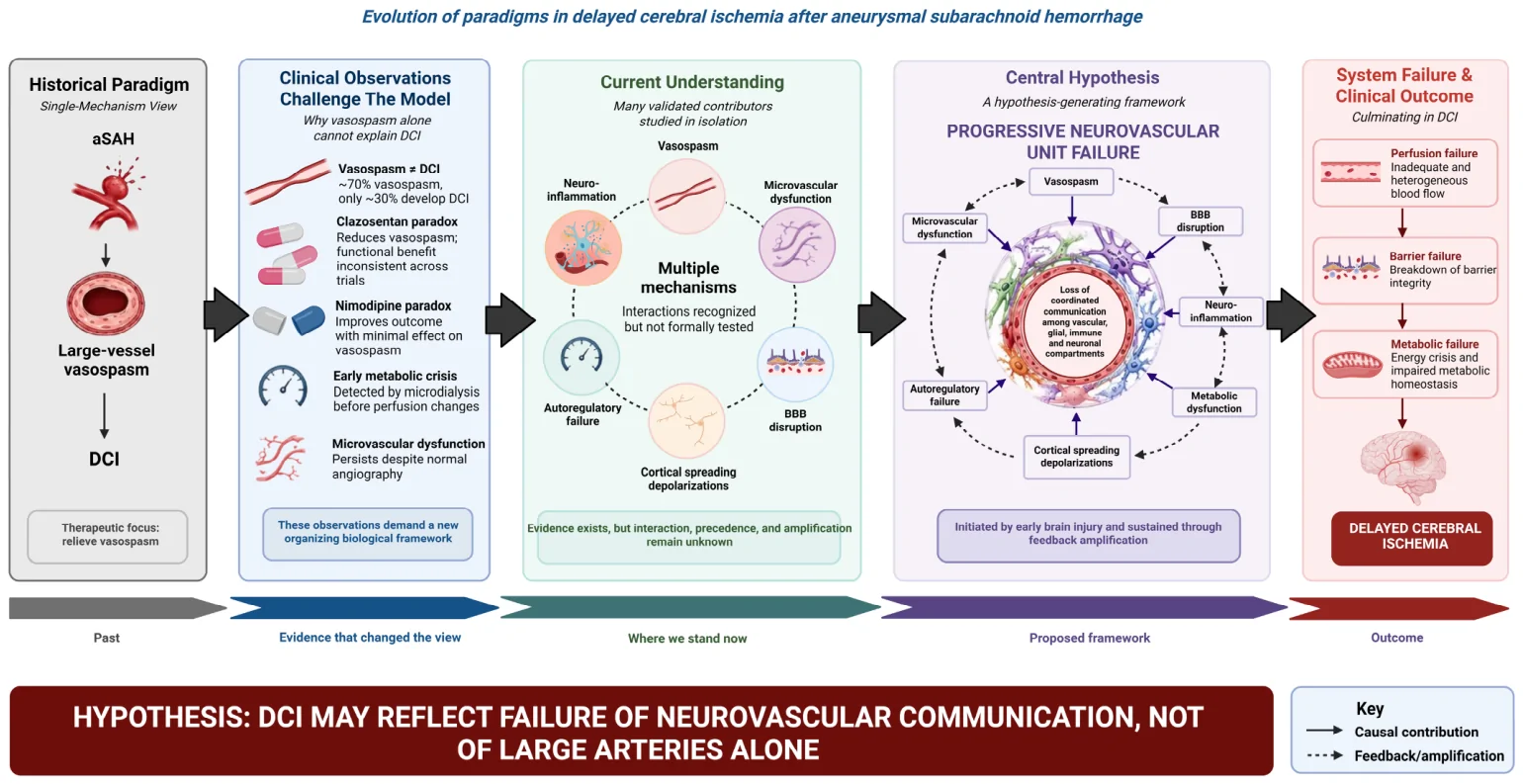
From Fragmented Mechanisms to an Integrated Conceptual Framework. Created in BioRender. Yaqinuddin, A. (2026) https://BioRender.com/rafedlf.

The central hypothesis of this review is that progressive neurovascular unit failure may represent a key underlying process in delayed cerebral ischemia, offering a systems-based framework that may help explain how established mechanisms of secondary brain injury converge to produce the clinical syndrome. On this view, DCI may be better understood as a disorder of neurovascular communication than of large arteries alone; how this framework differs from earlier multifactorial reviews is set out at the end of Section 6. The proposed conceptual framework is shown in Figure 1. 

## 2. Why Is the Neurovascular Unit an Appropriate Organizing Framework?

### 2.1. The Neurovascular Unit as a Functional Network

The neurovascular unit (NVU) is a multicellular partnership between neurons, astrocytes, endothelial cells, pericytes, smooth muscle cells, basement membrane, and immune cells. Emergent communication between these components maintains perfusion, barrier integrity, and metabolic balance. Neurovascular coupling, the best-studied function, was once described as a simple relay in which neurons signal astrocytes, and astrocytes signal vessels. That view has not held up. Coupling is now understood as a distributed, multidirectional process across capillaries, penetrating arterioles, and pial arteries. The mediators engage these vessels in a coordinated sequence of initiation, modulation, transmission, retrograde propagation, and implementation [10]. This sequence is carried out by the network’s connectivity, not a singular cell.

This connectivity goes beyond a classic anatomical unit. NVUs are variable along the arteriole–capillary–venule axis and across the brain, and neighboring segments coordinate with one another. For instance, in retrograde vasodilation, a signal that begins in capillaries can travel upstream to adjust the arterioles’ tone several branches away [11]. Thus, the functional unit can be defined as the communication pathway connecting segments, not the vessel segment itself.

### 2.2. Emergent Functions of the Neurovascular Unit

The core functions of the NVU arise from the collective networking of the comprising cells, not from any one cell acting alone. The integrity of the BBB relies on the combined contribution of endothelial tight junctions, pericyte control of transcytosis, and astrocytic maintenance of the basement membrane. The loss of any of these factors compromises the strength of another [10]. Autoregulation is driven by smooth muscle myogenic responses, with astrocytes and pericytes modulating capillary tone to fine-tune local blood flow [12]. Neurovascular coupling requires that the signal be initiated by neurons, relayed by astrocytes, and retrogradely propagated by the endothelium to upstream arterioles [10]. The coordinated action of barrier function, autoregulation, and coupling results in perfusion. Disruption of any of these components can limit flow, despite normal appearance of vessels. The output combination of barrier, flow, perfusion, and metabolic supply results in homeostasis.

Clinically, repairing a single component may not be sufficient to restore perfusion, barrier integrity, or metabolic supply, since no single element alone causes the problem. Reopening a constricted artery may improve conduit flow, but capillary-level regulation or barrier function is not necessarily restored, as these depend on communication pathways that the intervention does not reach [13,14]. In this context, delayed cerebral ischemia (DCI) can be viewed as consistent with a failure of coordinated output rather than with injury to a single structural component.

### 2.3. Defining Neurovascular Unit Failure

The progressive loss of coordinated communication among the vascular, glial, immune, and neuronal compartments, when it exceeds the network’s adaptability, can be termed NVU failure. The use of the term failure is intentional, since related terms describe narrower or earlier stages of the same process. Dysfunction implies impairment that can be compensated for by regulatory reserves, preserving the overall output. Injury implies structural damage to a specific cellular component without denoting that system-level performance has been lost. Disruption describes an isolated abnormality confined to a single pathway or interface. In contrast, failure denotes the point at which compensatory mechanisms are exhausted and coordinated neurovascular communication is unable to maintain the integrated system composed of perfusion, barrier integrity, and metabolic homeostasis. This is a threshold, not a severity gradient, and different compartments may cross it at different times, as the evidence below illustrates. Operationally, dysfunction is a weakened response that recovers, whereas failure is a response that stays absent or inverted, for example, when spreading depolarization causes prolonged hypoperfusion instead of hyperemia in vulnerable tissue, or when sustained neuronal activity constricts vessels in mice [15,16]. Possible markers in patients include prolonged spreading ischemia on electrocorticography with flow monitoring and falling glucose with a rising lactate-to-glucose ratio on microdialysis [16,17]. Thresholds for these measures have been proposed for predicting DCI, but to our knowledge, none has been validated as marking the transition from dysfunction to failure [17]. The value of this distinction lies in its potential to identify when impaired NVU function exceeds the capacity for compensation, rather than simply reflecting more severe dysfunction. Although candidate physiological and metabolic markers may help characterize this transition, prospective studies are needed to establish operational criteria for distinguishing failure from potentially reversible dysfunction. Until such criteria are validated, the distinction between compensated dysfunction and failure should be regarded as a conceptual proposal rather than an established category.

Different NVU functions seem to fail at different times. Three studies in male C57BL/6 mice, all using filament-perforation SAH, show this [15,18,19]. Within 3 to 4 h, pial and parenchymal arterioles no longer dilated to CO_2_, but neurovascular coupling (in vivo and in slices) and astrocyte endfoot calcium signaling were intact [19]. At 24 h, CO_2_ reactivity was still lost, and sustained stimulation caused parenchymal arterioles to constrict in vivo (forepaw stimulation) and in slices (electrical field stimulation), while the flow response to brief stimulation was preserved [15]. At one month, CO_2_ reactivity had recovered in parenchymal arterioles, but the response to brief stimulation was lost, and the response to sustained stimulation was reduced, though not significantly different from sham [18]. CO_2_ reactivity reflects chemoregulation of vascular tone and is distinct from pressure autoregulation, so these findings should not be taken as evidence about autoregulatory capacity. These time points come from separate groups of animals, since coupling cannot be followed in the same animal, and no similar course has been shown in patients, to our knowledge. This pattern fits different NVU functions failing at different times, not everything failing at once.

A similar phenomenon can be observed in coagulation. Cerebral microthrombi form within minutes of hemorrhage in rodent models, an early response proposed to be compensatory for the vessel injury, though this remains speculative. Reported timelines vary: some studies describe resolution within 48 h, while others report near-baseline levels by day 1, with burden peaking between days 2–5. A second, more variable wave later in the first two weeks has increasingly been attributed to platelet-leukocyte interactions and neutrophil extracellular traps, and was associated with infarction in human autopsy series and experimental studies [20]. This shift, from an early hemostatic response to a later, immune-driven pathology, reflects a threshold event within a compartment rather than a uniform collapse. This suggests functional dissociation may precede structural injury.

### 2.4. Can the NVU Serve as an Organizing Framework?

A critical way to evaluate this framework is to determine whether considering NVU failure as the initiating event, rather than as secondary to vasospasm, modifies evidence-supported predictions. A primary-NVU model would be expected to show three things: vasospasm-targeted therapy should improve outcome inconsistently across trials, endpoints, and populations, NVU-level disturbances should appear hours to days before DCI, and a network architecture should naturally generate the heterogeneity that single-mechanism models cannot explain.

The framework predicts that risk will not simply add up. Existing multifactorial models, in which several processes together produce a perfusion-metabolism mismatch, may also accommodate synergistic interactions, so non-additivity by itself does not distinguish the two views. The NVU framework is more specific about where such interactions should arise: if the effects were independent, DCI risk would rise gradually with each added abnormality [4]. A network running out of compensation instead implies a sharper rise when abnormalities in two compartments occur together in the same territory. This can be tested as an interaction in prospective monitoring studies. Additive risk, or vasospasm severity alone explaining DCI, would weaken the framework. This prediction provides a way to distinguish the proposed framework from purely additive models by testing whether interactions between established mechanisms contribute to deterioration beyond their individual effects. Whether such interactions occur and meaningfully influence DCI risk remains to be established.

One informative observation is the limited benefit of vasospasm-directed therapy. Suppressing vasospasm has not reliably improved outcomes. In CONSCIOUS-2 (clipping), clazosentan at 5 mg/h produced only a nonsignificant 17% relative-risk reduction (21% vs. 25%, *p* = 0.10), while poor Glasgow Outcome Scale-Extended (GOSE) outcome was actually slightly higher with treatment (29% vs. 25%) [13]. In CONSCIOUS-3 (coiling), the 15 mg/h dose did reduce the composite endpoint (27% to 15%; OR 0.474, 95% CI 0.275–0.818, *p* = 0.007), yet poor GOSE outcome again remained slightly more common (28% vs. 24%), and mortality was unchanged [14]. In contrast, two Japanese phase III trials of clazosentan at 10 mg/h reported benefit after both clipping and coiling, whereas REACT at 15 mg/h did not reduce clinical deterioration due to DCI [21,22]. Clazosentan benefit, therefore, varies by trial, endpoint, and population rather than being absent. Hemorrhage burden may help explain the gap between composite-endpoint benefit and functional outcome; a thick, diffuse clot predicts a far higher morbidity (36.1% vs. 14.7%) and poorer outcomes (32.7% vs. 16.2%) [23]. A pooled meta-analysis found that clazosentan reduced vasospasm-related infarcts and vasospasm incidence but did not significantly improve functional outcome or mortality [24]. One possible explanation is that clazosentan has minimal effect on concurrent microcirculatory dysfunction, including microthromboembolism, endothelial injury, and cortical spreading ischemia; this remains speculative [24]. Vasospasm and DCI co-occur only 30–80% of the time, and TCD carries its own well-known sensitivity and specificity limits [12].

If the wrong layer is being targeted by therapy, network disturbance should show up before DCI, and several studies report it. Microdialysis shows falling glucose and rising lactate-to-glucose ratio up to about two to three days before CT-confirmed infarction, with glutamate rising only later [17]. Serum and CSF vascular injury markers rise within the first day, and inflammasome proteins increase from day 1 [25,26]. In mice, coupling fails by 24 h, and to our knowledge, the corresponding timing in patients is unknown [15]. Electrophysiology follows the same logic: cortical spreading depolarization (CSD) triggers opposite vascular responses depending on network state. CSDs occur in 72–80% of aSAH patients, independent of radiographic vasospasm; healthy tissue shows hyperperfusion, and compromised tissue prolonged hypoperfusion [6,16]. In one cohort, CSD count was more closely associated with DCI than angiographic vasospasm or perfusion CT (OR 2.064, AUC 0.836). In a small cortical-electrode series (*n* = 18), CSD had a positive predictive value of 86% and a negative predictive value of 100%, compared with a TCD positive predictive value of 57% in a meta-analysis [6]. These signals are reported before clinical DCI, making them candidates for biomarker-guided intervention.

Patients with similar hemorrhage severity often diverge clinically in ways single mechanism models cannot explain. A network model predicts why: if the endothelial, microvascular, neuroimmune, and electrophysiological compartments need not fail at identical timing or severity, then the compartment that dominates should vary across patients, even with matched hemorrhage burden. This is examined in Section 4.2, where unresolved disagreement over whether pericytes drive or follow microvascular constriction is addressed as a candidate case of compartment-specific divergence rather than treated as a single, settled mechanism. Moreover, this logic is not unique to DCI, as we can notice a persistent NVU dysfunction in neurodegenerative disease where ongoing NVU abnormalities are seen after initial insult rather than any single pathway causing the chronic inflammation and progressive injury [27].

These findings, combined, suggest that the operative mechanism runs alongside large-vessel narrowing, appears before DCI’s clinical signature, and is only partially addressed by vasospasm-directed therapy. They do not prove that NVU failure is the main cause. Vasospasm may be one contributor within a failing network.

## 3. How Does aSAH Initiate Progressive Failure?

### 3.1. Early Brain Injury as the Initiating Insult

The first moments after aneurysm ruptures are thought to shape much of what follows. The rapid extravasation of arterial blood into the subarachnoid space results in an abrupt rise in intracranial pressure (ICP) [28,29]. This is followed by a fall in cerebral perfusion pressure and a transient period of global cerebral ischemia. This is not a focal injury but a shock to the network: all components of the neurovascular unit are simultaneously deprived of substrate, while its structural integrity is challenged by mechanical distortion. This insult is soon followed by oxidative stress, as ischemia-reperfusion and the earliest blood breakdown products generate reactive oxygen species that damage the lipids, proteins, and DNA of the endothelium, glia, and neurons [28,30,31]. Mitochondria are at the heart of both energy provision and oxidative stress, are injured early, and become self-perpetuating amplifiers of stress rather than passive casualties. Damaged mitochondria produce more reactive oxygen species, which further compromise mitochondrial function and extend cellular injury long after the hemodynamic event has resolved [32]. In the first hour, mechanical shear from the hemorrhage itself disrupts capillary architecture and astrocytic-endothelial contact [28,29].

Despite the severity of this early insult, these events alone may be insufficient to explain the development of delayed cerebral ischemia. Trials of vasospasm-directed therapy, long believed to target the dominant late mechanism, have not consistently improved functional outcome, highlighting a disconnection between local vascular narrowing and the clinical syndrome it was thought to explain [13,14,24]. This discrepancy suggests that EBI alone may not be sufficient to cause DCI, but may prime a network whose capacity for coordinated regulation is already impaired. We propose that this predisposes to progressive neurovascular unit failure rather than a fixed downstream event [28]; this remains a hypothesis supported mainly by experimental and observational data. This raises the next question: what sustains and propagates this initial network destabilization?

### 3.2. Blood-Derived Toxicity Produces Persistent NVU Stress

We propose that sustained exposure is a key contributing mechanism. Once in the subarachnoid space, hemoglobin, liberated iron, thrombin, and activated complement are not cleared instantaneously but remain in contact with the endothelial–glial interface for days, leading to persistent oxidative and proteolytic stress rather than a single toxic exposure [29,30,33,34]. Iron-catalyzed radical generation, thrombin-mediated receptor signaling on vascular cells, and complement-driven immune priming are mechanistically distinct but functionally converge on the same target: the degradation of the tight junctions and signaling architecture that allow the BBB to function as a regulated interface rather than a passive barrier [33,34,35]. This is consistent with BBB permeability increasing progressively over the early post-hemorrhage period rather than appearing and resolving as a single event; the interface is continually stressed, not struck once [33,35]. Clinically, this persistent toxicity may help explain why interventions aimed at any single blood-derived mediator have shown limited translational success: targeting iron, or thrombin, or complement individually addresses one input into a network-level problem rather than the problem itself [33,34]. The unresolved question is how this sustained, multi-input stress is organized into the specific, reproducible sequence of dysfunction that culminates in DCI.

### 3.3. Establishing the Biological Hierarchy

We propose that this progression from sustained network stress to delayed cerebral ischemia follows a hierarchical order. EBI is the inciting event, a synchronous shock involving ischemia, pressure elevation, oxidative and mitochondrial injury, and mechanical trauma [28,29,32]. This shock is propagated by a primary network disturbance: endothelial dysfunction and microvascular dysregulation, clinically manifested as impaired autoregulation, microthrombosis, and loss of neurovascular coupling, independent of large-vessel narrowing [36,37,38]. This disturbance is then compounded by BBB disruption, neuroimmune activation, and ongoing oxidative stress, which maintain and extend microvascular compromise, rather than being separate complications [33,34,35,38]. The final common pathway is progressive neuronal metabolic failure: neurons lacking dependable vascular and glial support lose the ability to maintain oxidative phosphorylation and ionic gradients, which clinically presents as delayed neurological decline, diagnosed as DCI [32,38]. This model does not replace vasospasm, inflammation, or cortical spreading depolarization, which are all real and clinically relevant processes [28,37,38]. What the framework proposes to change is their explanatory status, not as competing causes of DCI but as possible manifestations of a single underlying process: progressive failure of neurovascular communication initiated by EBI and sustained by ongoing blood-derived toxicity. Importantly, this hierarchy is inferred from studies of individual components, many in experimental models and examined at different time points; it has not been demonstrated as a continuous causal sequence in patients.

## 4. How Does Progressive NVU Failure Produce DCI?

### 4.1. Primary Network Disturbance: Endothelial Dysfunction

Endothelial dysfunction after aSAH is not a localized injury. It is the disruption of a signaling relay that the rest of the NVU relies on. In health, the endothelium transduces capillary-level potassium changes into hyperpolarization by retrogradely conducting vasomotor signals. The hyperpolarization propagates through gap junctions to control tone in arterioles several branch points upstream [10]. This relay function explains why endothelial injury produces effects that outlast and outrun the injured segment itself.

Several distinct insults occur at the onset of disruption, compounding over the first hour to the first day. These include loss of nitric-oxide-mediated signaling, degradation of the glycocalyx, and oxidative injury to the endothelial cells themselves [12,39]. These converge on vasomotor conduction and barrier integrity. Instead of each compartment getting injured on its own, evidence suggests that a single upstream failure propagates across them.

Since the endothelium serves as both a barrier and a signaling relay, it is reasonable to expect the two to fail together, with damage spreading beyond the segment that initially made contact with blood. The same retrograde pathway that normally carries a healthy signal upstream does not distinguish a healthy signal from a failing one [10].

### 4.2. Primary Network Disturbance; Microvascular Dysregulation

Loss of coordinated control over vessel diameter at the capillary and arteriolar level defines microvascular dysregulation. In normal conditions, this control depends on continuous signaling among pericytes, smooth muscle, and the endothelium. At the arteriolar level, smooth muscle contributes independently of the capillary-bed pericyte debate: after SAH, smooth muscle cells undergo phenotypic transformation and migration, which we propose may add to calcium-driven constriction [12].

The mechanism behind the breakdown of capillary-level control remains actively disputed, and the debate itself is revealing. Three lines of evidence directly conflict: one argues that pericyte calcium dysregulation drives sustained constriction; another links capillary narrowing to pericyte apoptosis rather than active contraction; and a third reports reduced capillary perfusion despite stable pericyte density and diameter, implying a passive deficit downstream of arteriolar constriction instead of a pericyte-mediated process [39,40,41]. This review does not attempt to reconcile these differences; in a system with overlapping failure modes, different groups will naturally obtain discordant findings depending on the time points or compartments they sample, and the interpretations may be compatible with progressive NVU failure.

What is not contested is that microvascular constriction and microthrombosis occur in close temporal and spatial proximity, with microthrombi forming within minutes and aligning with arteriolar constriction; whether one causes the other or both share a trigger remains unclear [20]. This matters because the process may operate largely independently of the timeline of angiographic vasospasm, which is why microcirculatory dysfunction has been proposed as a more direct correlate of tissue perfusion than large-vessel narrowing [12,24]. Whether rescuing a single compartment is sufficient remains unknown; no study has directly compared pericyte-targeted and arteriole-targeted interventions in the same aSAH model.

### 4.3. Network Amplification: Blood–Brain Barrier Failure

BBB failure seems to amplify ongoing disturbances rather than initiate them. It converts localized dysfunction into a whole-brain problem by allowing unregulated exchange between blood and parenchyma. Its timing is consistent with this amplifying role. Substantial disruption appears early in the injury course. Tight-junction proteins are degraded at the same early time point at which oxidative stress affects endothelial and pericyte compartments [39]. Mechanistically, barrier failure appears to follow the same inflammatory signaling cascade triggered by injury to the endothelium and glycocalyx. Endothelial and astrocytic pathways converge on a shared degradative process despite having distinct upstream triggers [12,42].

Once the barrier is compromised, its failure feeds back onto affected compartments through two routes. Chemically, albumin extravasation disrupts astrocytic potassium buffering. This produces neuronal hyperexcitability and creates a direct link between vascular failure and neuronal signaling. Mechanically, albumin efflux raises interstitial oncotic pressure. This drives vasogenic edema on top of existing cytotoxic edema. The resulting tissue pressure may further compress the microvasculature, acting as a physical amplifier to the biochemical one [12]. Together, these processes appear to increase metabolic demand precisely when supply is least able to meet it.

### 4.4. Network Amplification: Neuroimmune Feedback

Neuroimmune signaling seems to act as a second amplifying mechanism. In contrast to the barrier-related processes, it appears to rely on a two-way loop between astrocytes and microglia rather than on direct chemical escalation. The interaction seems to build over the first few days after the hemorrhage, supported by ongoing cross-talk between the two cell types.

Damage-associated molecular patterns released from injured or lysed cells bind receptors on both microglia and astrocytes at roughly similar times. Microglia often respond first, within minutes. Their early activity tends to influence astrocytes before producing any direct neuronal effects. One such example is HMGB1: it increases aquaporin-4 expression in astrocytes, but only when microglia provide IL-1β, suggesting a relay, not two independent responses to the same signal. Once activated, astrocytes signal back to microglia, increasing MMP-9 expression, contributing to barrier changes. A complement-based loop runs alongside the cytokine cycle. Microglia-derived C1q shifts astrocytes toward a more neurotoxic phenotype. Those astrocytes then produce C3, which acts on microglia via the C3–C3aR pathway, thereby increasing activation. The structure mirrors the cytokine loop but uses a different signaling axis. This complement activity also extends into the vascular compartment. Elevated CSF C3a has been linked to delayed ischemic deficits through activation of coagulation pathways, connecting neuroimmune amplification to microthrombosis and perfusion disturbances [42].

These reciprocal loops reinforce one another. Each cycle increases the next round of glial activation and further weakens the barrier that would otherwise limit the spread of injury. This pattern is not limited to rodent models. Markers of vascular injury and inflammasome activation increase in serum and CSF starting on day 1 after hemorrhage, and an association with clinical course has been reported in four cohorts from three countries (UK, Germany, and Spain), although samples are small and biomarker panels and outcome measures differ [25,26,43,44]. In a retrospective 82-patient cohort, CSF IL-6 was associated with DCI-related infarction but not with angiographic vasospasm [8]. The lack of correlation suggests that at least part of the neuroimmune response may progress independently of large-vessel narrowing rather than merely tracking it.

### 4.5. Final Common Pathway: Neuronal Metabolic Failure

The disturbances described above point to neuronal energy failure; to our knowledge, no single study has followed the entire progression from network disruption to metabolic collapse. At its core, this endpoint reflects neurons losing the ability to maintain their ion gradients when the damaged network can no longer meet the energetic demand. Human cerebral microdialysis shows this clearly: extracellular glucose falls, and the lactate-to-glucose ratio rises more than 2 days before CT can detect an infarction [17]. Glutamate increases later, marking the shift from a supply–demand imbalance toward more severe energy failure.

This transition is not only biochemical. It also has an electrophysiological component. Cortical spreading depolarization (CSD), a self-propagating wave of near-complete neuronal depolarization, is common in patients with aSAH. In healthy tissue, CSD produces a brief hyperperfusion response. In tissue where neurovascular coupling is already compromised, it produces spreading ischemia instead, a prolonged hypoperfusion that worsens injury [16]. The wave drives constriction through potassium-mediated smooth muscle depolarization and simultaneously suppresses nitric oxide production, removing the vasodilatory signal that would normally counterbalance it [6]. Whether CSD ends up protective or harmful depends on how intact the surrounding network was beforehand. The same ionic collapse also releases glutamate, initiating the excitotoxic cascade.

The excitotoxic pathway itself is well described. Glutamate activates NMDA receptors, leading to calcium overload and downstream lethal signaling. Selective GluN2B antagonism reduces neurological deficits following experimental hemorrhage and lessens the severity of vasospasm, suggesting that glutamatergic signaling may influence vascular responses [45]. Even this pathway, often treated as the final step, remains connected to the broader network.

A second cohort supports the clinical relevance of metabolic failure. In 49 SAH patients monitored with cerebral microdialysis, investigators recorded 113 minor and 42 major lactate-to-pyruvate ratio events over a median of six days. Intervention restored metabolic balance in most cases, and DCI-related infarction occurred in only 10 percent of patients, with just two infarcts appearing in monitored regions [46]. The sequence differs from Tholance et al., but the conclusion is similar: metabolic derangement is common, detectable, and potentially modifiable before infarction is established [17].

Neuronal death beyond this point takes several forms. Ferroptosis is particularly well studied in this setting. In murine models, it affects endothelial cells and microglia as well as neurons, and inhibiting it preserves tight junctions [47]. Different cell populations follow distinct routes depending on timing and upstream stressors, but the endpoint remains the same: the loss of ionic gradient maintenance.

**Figure 2:**
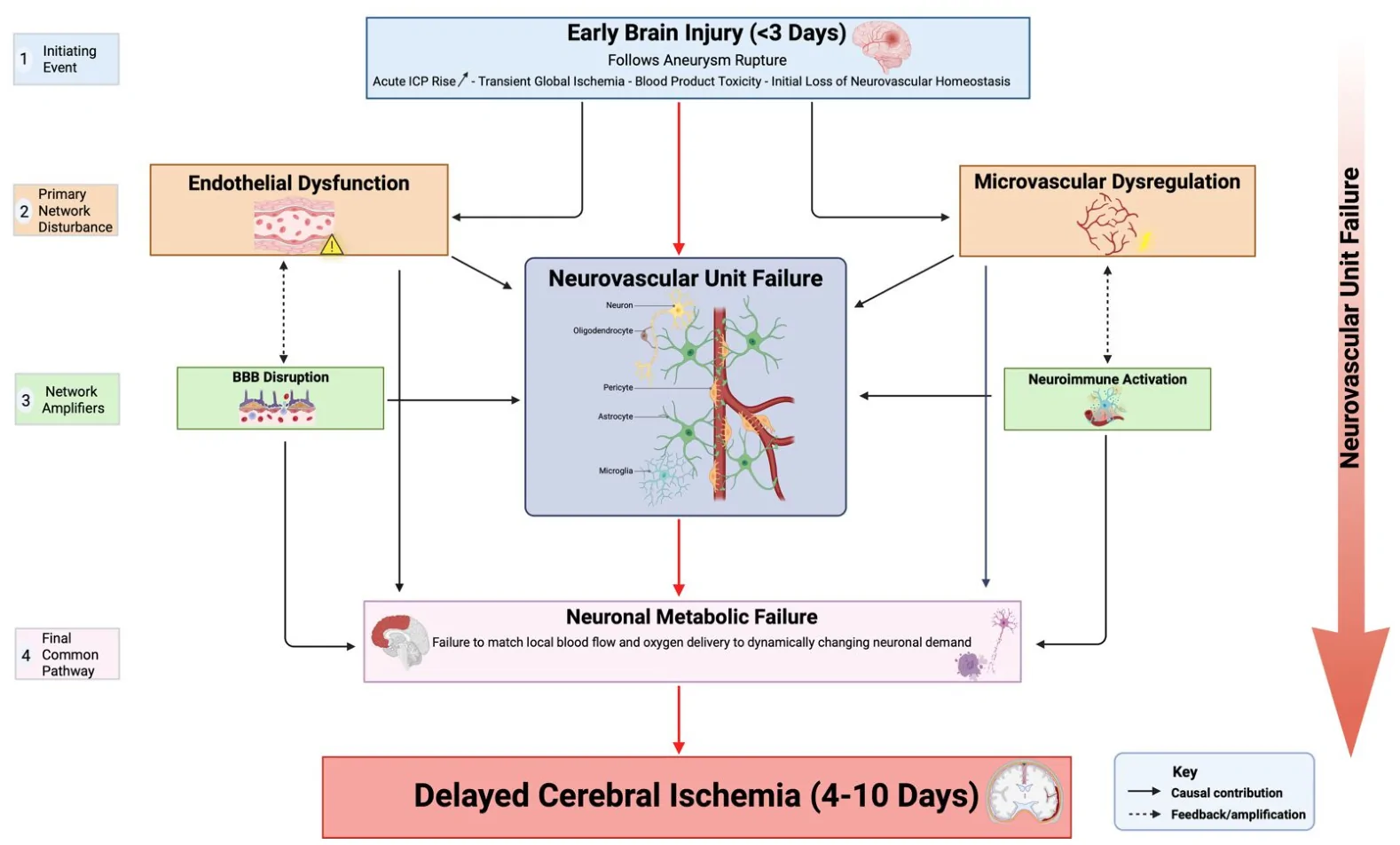
Progressive Neurovascular Unit Failure After aSAH. Created in BioRender. Yaqinuddin, A. (2026) https://BioRender.com/rvaztkp.

These processes may form a single trajectory, although this has not been shown as a continuous sequence in patients. Endothelial and microvascular disturbances are proposed to initiate the failure, with barrier breakdown, spreading depolarization, and neuroimmune signaling amplifying it. Neuronal energy failure is where these processes converge. Each part of the sequence describes a different cell population caught within the same deteriorating network. The key mechanisms contributing to NVU failure after aSAH can be found in Figure 2.

## 5. Can the NVU Framework Help Interpret the Clinical Features of DCI?

The previous section explored the mechanistic basis through which progressive NVU failure may culminate in delayed cerebral ischemia. So, this section will focus on determining if this framework can actually translate clinically, particularly the observations that have challenged traditional disease models.

### 5.1. Reconsidering the Necessity and Sufficiency of Vasospasm

This framework offers one possible interpretation of a central clinical challenges in DCI: the dissociation between angiographic vasospasm and the development of ischemia. Historically, vasospasm was regarded as the principal cause of DCI, and the two were often treated as closely equivalent disease processes [48,49]. However, this assumption was fundamentally disrupted when clinical observations showed that substantial angiographic vasospasm can occur without ischemia, whereas DCI can develop despite little or no large-vessel narrowing [50,51]. This raised questions about the relationship between vasospasm and DCI. The proposed framework addresses this uncertainty by positioning vasospasm as one contributor to progressive NVU failure rather than as the defining disease process [52,53]. Within this framework, the apparent paradox is no longer unexpected, because different combinations of vascular, microcirculatory, inflammatory, barrier, and metabolic disturbances may drive the system toward the same endpoint of neuronal metabolic failure. Vasospasm may therefore contribute to DCI as part of cumulative network dysfunction, but its presence alone does not determine whether clinical ischemia will develop.

### 5.2. Why Macrovascular Treatment Has Limited Success

On that same note, the clinical dissociation between vasospasm and DCI also helps explain another long-standing challenge in aSAH research: the limited success of therapies directed solely at large-vessel vasospasm. For decades, the assumption that vasospasm was the principal driver of DCI led to the development of therapies aimed primarily at restoring large-artery caliber. Although several interventions successfully reduced angiographic vasospasm, their effects on delayed cerebral ischemia and long-term functional outcomes were often modest or absent [13,53]. Similarly, the STASH trial found that statins did not improve functional outcomes or reduce complications after aSAH [54]. This therapeutic discordance further challenged the concept that vasospasm alone defines the disease process. However, within an NVU-centered model, correcting arterial narrowing addresses only one component of progressive NVU failure, while dysfunction across the wider neurovascular system may persist. This rationale is further supported by nimodipine, the current standard of care, which improves neurological outcomes despite having only modest effects on large-vessel vasospasm [48]. Its clinical benefit is thought to reflect broader actions on neurovascular function, which could be viewed as being consistent with the proposed model and the multifactorial pathophysiology of DCI [53].

### 5.3. Delayed Onset and Clinical Heterogeneity

A further clinical feature addressed by the NVU framework is the variable timing and presentation of DCI. Its delayed onset is more consistent with progressive system failure than with an immediate, isolated event [55]. Within the proposed framework, this delay reflects both the time required for disturbances to develop across the NVU and the system’s temporary capacity to compensate for their effects. For example, neuroinflammation evolves progressively as circulating leukocytes are recruited and the microglial response becomes established, allowing inflammatory injury and its effects on endothelial, microvascular, and metabolic function to accumulate over time [42,52]. Meanwhile, autoregulatory adjustments, collateral perfusion, metabolic reserve, and restoration of ionic gradients may initially preserve substrate delivery and neuronal function, thereby delaying the clinical expression of the underlying dysfunction [17]. DCI emerges when the growing neurovascular burden exceeds this compensatory capacity and the NVU can no longer maintain an adequate balance between cerebral substrate delivery and neuronal metabolic demand [53]. Clinical microdialysis findings showing that disturbances in cerebral energy metabolism may precede radiographic hypoperfusion and infarction by up to two to three days are consistent with this sequence, as they indicate a period of progressive metabolic decompensation before irreversible tissue injury becomes established [7,17]. By placing these temporal disturbances within a compensatory system, the NVU framework may account for the delayed onset of DCI.

The same progressive and threshold-dependent process may also explain the clinical heterogeneity of DCI. Patients may differ in both the burden of NVU dysfunction and their capacity to compensate for it [56]. Differences in collateral circulation, autoregulatory capacity, metabolic reserve, and susceptibility to secondary injury may allow a similar pathological burden to remain clinically silent in one patient while producing earlier or more severe deterioration in another. Heterogeneity may also reflect variation in the dominant pattern of NVU dysfunction, as different contributing processes have been associated with distinct pathological and radiographic expressions of injury [57]. Taken together, these differences can be interpreted as variable expressions of progressive NVU failure, although existing multifactorial models can also accommodate them.

## 6. How Do NVU Frameworks Integrate Existing Paradigms?

The pathophysiological understanding of DCI following aSAH has evolved predominantly from large vessel vasospasm-centered towards a multifactorial framework which comprises microcirculatory impairment, neuroinflammation, blood–brain barrier disruption, cortical spreading depolarization, and metabolic disturbances [51,53]. Clinical trials investigating clazosentan, a selective endothelin-A receptor antagonist, which represents the complexity between angiographic vasospasm and neurological outcomes, were not able to demonstrate significant benefits that translate into improved functional outcomes [13,14,24].

In contrast, two randomized Japanese phase III trials demonstrated significant clinical benefits of clazosentan when administered at 10 mg/h following either surgical clipping or endovascular coiling. The incidence of vasospasm-related morbidity and overall mortality decreased from 39.6% to 16.2% in the clipping study and from 28.8% to 13.6% in the coiling study. A predetermined pooled analysis also showed decreases in overall morbidity and adverse functional outcomes on the modified Rankin Scale (mRS) [22]. However, the REACT trial, which evaluated clazosentan at 15 mg/h in patients with thick, diffuse subarachnoid hemorrhage receiving standard care including nimodipine, did not demonstrate a significant reduction in its primary endpoint of clinical deterioration due to DCI (15.8% versus 17.2%; *p* = 0.734) [21].

These apparently conflicting findings should be interpreted considering the methodological and clinical differences present in the studies. The CONSCIOUS trials and the Japanese phase III trials assessed a comparable primary endpoint framework related to vasospasm-associated morbidity and overall mortality within 6 weeks. In the Japanese trials, all-cause morbidity and mortality were assessed as a second primary endpoint. In contrast, REACT concentrated on clinical decline due to DCI, assessing cerebral infarction and long-term functional results independently. The trials varied in regional demographics, procedures for securing aneurysms, clazosentan dosages (5, 10, or 15 mg/h), timing for starting treatment, standard care, and eligibility requirements concerning hemorrhage severity. Specifically, nimodipine is not commonly employed as standard treatment in Japan, while it was utilized in REACT. These variations may have influenced the inconsistency in treatment results, although the specific impacts cannot be definitively established [13,14,21,22].

Overall, these results indicate that addressing endothelin-induced vasospasm can reduce significant ischemic complications in specific groups, although its impact on overall neurological recovery varies across studies. Thus, the lack of functional advantage in specific trials should not be seen as evidence that macrovascular constriction is irrelevant in DCI development. Instead, the varied therapeutic responses align with a complex disease process where macrovascular dysfunction interacts with microvascular, inflammatory, electrophysiological, and metabolic issues. In the suggested neurovascular unit (NVU) model, vasospasm is viewed as a significant, potentially modifiable factor in secondary injury rather than the sole cause of DCI.

The NVU framework does not discard historical models; rather, it proposes the NVU as a biological architecture in which these mechanisms may intersect. Rather than functioning as a distinct competing hypothesis, NVU dysfunction may represent the common pathway through which vascular, cellular, and metabolic insults interact to cause tissue injury. The integrated historical paradigms are presented in Table 1.

Relation to previous reviews Earlier comprehensive reviews, notably Budohoski et al. [51], and later Geraghty and Testai [53], Dodd et al. [48], and Stragier et al. [49], established that DCI is multifactorial and described its contributing mechanisms, and Solár et al. [62] examined the BBB and NVU in SAH at the molecular level. The present framework does not add new mechanisms. It may differ in emphasis in four respects: (i) it proposes an operational distinction between compensated NVU dysfunction and NVU failure, with candidate markers in patients (Section 2.3); (ii) it specifies where non-additive interactions between NVU compartments would be expected and how they could be tested, while recognizing that existing multifactorial models can also accommodate synergy (Section 2.4); (iii) it organizes biomarkers by the NVU compartment they reflect rather than by assay type (Section 7.1, Table 2); and (iv) it states prespecified predictions under which it would be revised or rejected (Section 7.3). Whether these features improve prediction or treatment remains to be tested. Accordingly, the present work should be read as a hypothesis-generating perspective rather than as an established disease model.

## 7. How Does This Framework Change Clinical Practice and Future Research

If the hypothesis that DCI reflects progressive neurovascular unit failure, rather than a delayed arterial event alone, is supported, then activities that depend on how the disease is conceptualized, including biomarker design, therapeutic strategy, and hypothesis generation, may benefit from being reorganized around the NVU as the unit of analysis, not around individual cell types or individual mechanisms.

### 7.1. Biomarker Development

Under the conventional model, biomarkers are typically grouped by assay type: imaging markers, serum protein markers, electrophysiological markers, and cerebrospinal fluid markers are studied and reported as separate literatures [64,65]. This organization is a direct consequence of treating vasospasm, inflammation, and neuronal injury as independent processes, each deserving its own biomarker program. If NVU failure is a unifying process, as proposed here, biomarkers should be organized according to the neurovascular compartment they interrogate, like endothelial and microcirculatory function, blood–brain barrier integrity, glial–neuroimmune signaling, and neuronal metabolic status, rather than by the laboratory technique used to measure them. A single compartment can then be interrogated by multiple modalities simultaneously: microcirculatory dysfunction captured by both perfusion imaging and circulating endothelial markers, or BBB breakdown captured by both permeability imaging and serum barrier proteins [65,66]. This reframing has an immediate practical consequence: no single biomarker, however sensitive, should be expected to reliably predict DCI, because of the possibility that no single compartment failure may be sufficient to produce the syndrome. Multimodal phenotyping, combining markers from at least two compartments, ideally with serial sampling that captures the transition from initiation to amplification, is therefore a natural implication of the framework rather than a purely methodological refinement: biomarker studies would be more informative if they specified which NVU compartment a given molecule reflects [64]. Whether panels built on this compartment-based logic actually outperform single-compartment markers in practice is a distinct empirical claim, and it is stated explicitly once as one of the framework’s testable predictions in the following section.

### 7.2. Therapeutic Reorientation

The clinical trial record in DCI is dominated by single-target interventions, calcium channel blockers, endothelin receptor antagonists, magnesium, statins, each designed to interrupt one node of the cascade, typically vasospasm [13,14,24,54]. Their modest and inconsistent efficacy may reflect, at least in part, intervention on a single output of a multi-component process, although trial design, patient selection, timing, and dosing may also contribute. If EBI initiates a process of reciprocal endothelial–glial–neuronal decompensation that amplifies through feed-forward loops, then blocking arterial narrowing alone may not restore coordinated NVU function, because vasospasm may be a downstream manifestation rather than the origin of the failure. This reframing suggests that future therapies may need to adopt combination strategies aimed at restoring communication across NVU compartments—for example, pairing early endothelial and barrier stabilization with downstream neuroimmune modulation and hemodynamic support—rather than escalating the dose or specificity of a single-pathway drug [37,67]. It also reorients the timing question: because the framework proposes that the decisive injury occurs at initiation, not at the moment vasospasm becomes angiographically visible, interventions delivered in the early brain injury window should, in principle, carry more preventive value than those delivered once microcirculatory and barrier failure have already propagated. This does not diminish the clinical importance of vasospasm treatment or hemodynamic augmentation; it repositions them as management of a late network consequence rather than as management of the disease process itself [67].

### 7.3. Testable Predictions and Future Research

A framework earns scientific standing only if it generates predictions that could, in principle, prove it wrong. This framework generates five such predictions, each traceable to a specific stage of the proposed hierarchy:

1. Abnormal endothelial and BBB markers measured within 24–72 h of rupture should precede clinical or radiographic DCI by at least 24 h in most patients who develop DCI. A vasospasm-centered model would instead predict that these markers track angiographic vasospasm severity [64,65,66].
2. A panel combining markers from at least two NVU compartments should show significant incremental discrimination over the best single-compartment marker (for example, an increase in AUC or a net reclassification improvement) in a prespecified validation cohort [64,68].
3. Serial multimodal data should identify reproducible NVU phenotypes (for example, endothelial-, barrier-, or neuroimmune-predominant) that predict outcome independently of hemorrhage burden [23].
4. Phenotype-guided combination therapy targeting at least two compartments should outperform single-target treatment on a prespecified DCI or functional endpoint in adequately powered trials [37,50].
5. Within the same vascular territory, concurrent abnormalities in two NVU compartments (for example, an endothelial or barrier marker together with clustered spreading depolarizations or a microdialysis metabolic event) should increase DCI risk more than the sum of their separate effects, detectable as a significant interaction term in prospective multimodal monitoring studies; purely additive effects would argue against the framework (Section 2.4).
6. Not all of these predictions are unique to the NVU framework. Predictions 1–3 are also compatible with existing explanations of DCI, including early brain injury and multifactorial models; confirming them would be consistent with, but would not specifically support, progressive NVU failure. Prediction 4 is likewise expected under multifactorial models. Prediction 5 is the most discriminating, because it specifies compartment- and territory-specific interactions; even so, it would test a specific expectation of the framework rather than establish NVU failure as a distinct disease model.

**Figure 3:**
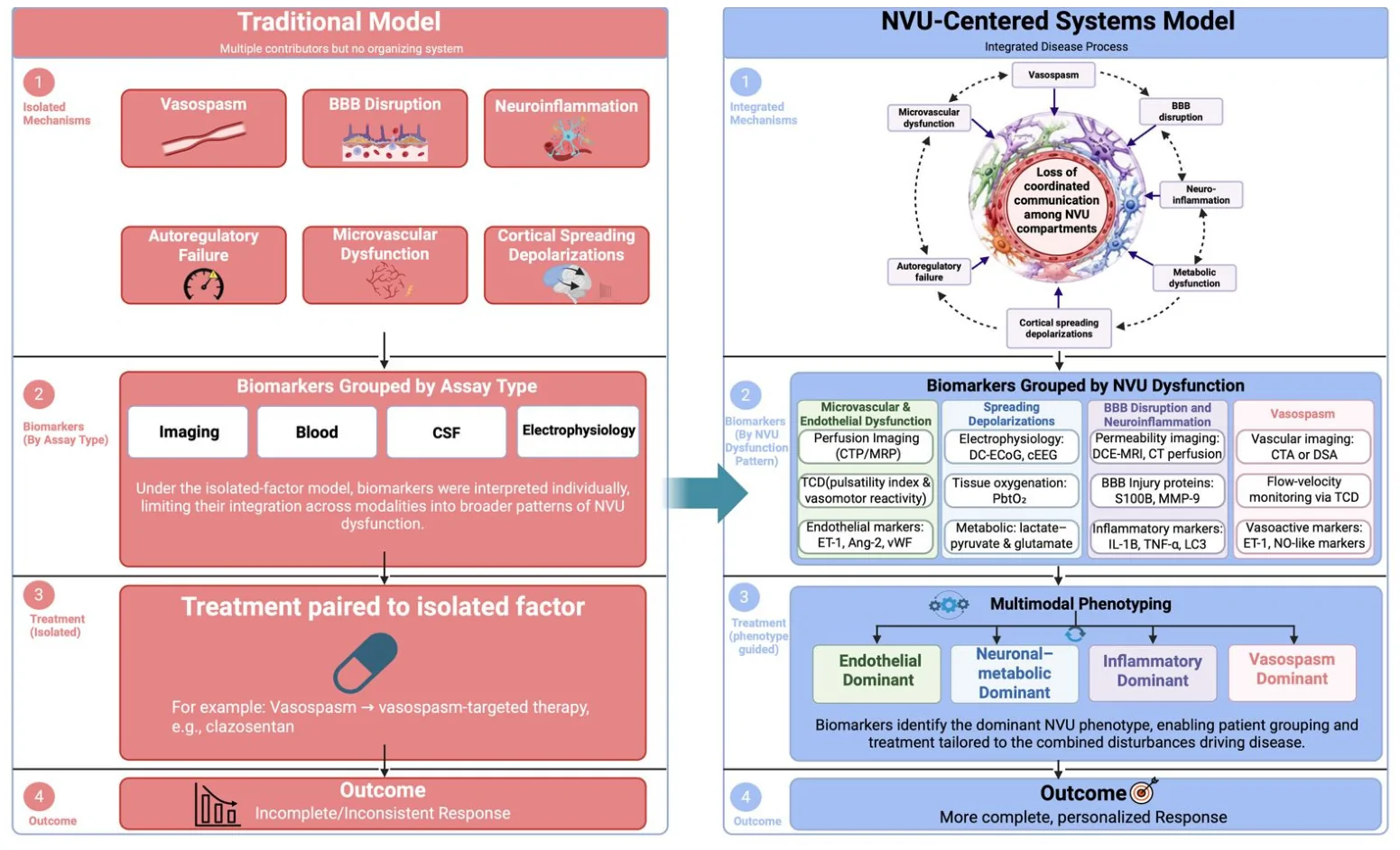
From Mechanism-Based Treatment to Neurovascular Unit Restoration. Created in BioRender. Yaqinuddin, A. (2026) https://BioRender.com/rehx5s6.

Each of these predictions is falsifiable: if early microcirculatory and barrier dysfunction do not reliably precede DCI, if combined panels add no predictive value over single markers, if no reproducible NVU phenotypes emerge, if abnormalities in different compartments combine only additively, or if combination therapy fails to outperform single-target treatment in adequately powered trials, the framework would need to be revised or abandoned. In short, the value of this framework lies in the questions it generates rather than in its explanatory scope; it is offered as a hypothesis to be tested and should be revised or abandoned if these predictions fail. The mechanism-based treatment for NVU restoration is summarized in Figure 3, and NVU biomarkers and therapeutic targets are listed in Table 2.

**Table 2:** Neurovascular Unit-Based Biomarkers and Therapeutic Targets.

NVU Compartment/Disease Stage	Representative Biomarkers	Biological Function	Clinical Relevance	Therapeutic Target/Intervention	Translational Maturity	References
Initiation—Early Brain Injury	S100B, GFAP, UCH-L1; ICP/CPP-derived metrics	Marks the overall severity of the initial injury—the earliest sign of network stress, before any single compartment starts to fail	Flags at-risk patients within hours of hemorrhage; higher levels track with worse early injury and worse long- term outcome	Early aneurysm repair; keeping ICP/CPP in target range; avoiding further insults during this window	Moderately mature—S100B and GFAP are validated across cohorts, but not yet used in real time to guide compartment-specific treatment	[50,64,65]
Primary Network Disturbance—Endothelium and Microcirculation	von Willebrand factor, endothelin-1, soluble thrombomodulin, microthrombosis markers; perfusion CT imaging; brain tissue oxygen reactivity; TCD velocities (indirect large-vessel surrogate, not a direct measure of microcirculatory function)	Reflects activation of the vessel lining, loss of normal vessel tone, and the earliest small-vessel clotting and narrowing	Signals the shift from early brain injury toward delayed ischemia—the first compartment where network failure becomes measurable	Endothelin receptor blockade (clazosentan); nimodipine, blood pressure/volume management, antiplatelet therapy	Advanced clinical evaluation: clazosentan demonstrated reductions in vasospasm-related complications in clinical trials, although findings differed across studies. CONSCIOUS-2 did not demonstrate significant improvement in its primary clinical endpoint, while CONSCIOUS-3 demonstrated a reduction in its primary composite endpoint at the higher dose without improvement in functional outcomes. Two Japanese phase III trials reported significant reductions in vasospasm-related morbidity and all-cause mortality composite endpoints, supporting approval in Japan. However, the international REACT trial did not demonstrate a statistically significant reduction in clinical deterioration due to DCI.	[13,21,22,50,64,65,67]
Amplification—Blood–Brain Barrier & Neuroimmune Feedback	MMP-9, claudin- 5/occludin fragments, ICAM-1, VCAM-1, IL- 6, IL-1β, and related inflammatory cytokines (e.g., IP-10, MCP-3, CX3CL1)	The vessel barrier breaks down, triggering a self-reinforcing wave of inflammation that spreads outward from the original endothelial injury	Tracks with brain swelling and DCI severity; the stage where single-pathway treatments have most often failed in trials	Statins (simvastatin); complement-targeted and anti-inflammatory strategies; conditioning-based therapies	Early clinical stage — simvastatin was tested in a large Phase 3 trial (STASH) with no benefit alone; other agents remain investigational	[37,54,64,65,66]
Final Common Pathway—Neuronal Metabolic Failure	NSE, tau; microdialysis lactate/pyruvate ratio; cortical spreading depolarization on electrocorticography/EEG	Marks neuronal metabolic distress once upstream compartments can no longer compensate. NSE and tau reflect established neuronal injury; lactate/pyruvate ratio and spreading depolarizations indicate risk of tissue loss but do not by themselves establish irreversibility	Reflects metabolic distress and, for NSE and tau, injury that has often already occurred; the least treatable stage, but the one most directly tied to outcome. Proposed markers of the transition from compensated dysfunction to failure: a sustained rise in lactate/pyruvate ratio, or clustered or prolonged spreading depolarizations with spreading ischemia. Thresholds are not standardized, and irreversibility requires confirmation by imaging of infarction.	Ketamine (NMDA receptor blockade of spreading depolarization); cilostazol	Early-stage but mechanistically well supported—both agents remain single-center/pilot- level and are not yet standard of care	[6,7,17,52,65,69]

## 8. Limitations and Future Validation

Several limitations should be considered when interpreting the proposed NVU framework. A major limitation is that, although the available evidence demonstrates that endothelial dysfunction, microvascular dysregulation, BBB disruption, neuroimmune activation, and metabolic abnormalities occur after aSAH, the proposed temporal hierarchy linking these processes remains incompletely validated in humans. Much of the mechanistic evidence is derived from experimental models or observational studies examining individual components at different time points. Consequently, temporal association does not necessarily establish causality, and it remains uncertain whether the proposed sequence of early NVU disturbance, progressive amplification, and metabolic decompensation represents a consistent trajectory across patients or a pattern restricted to specific biological phenotypes.

Importantly, the failure of vasospasm-directed therapies to reliably improve functional outcome supports the insufficiency of vasospasm as a standalone model of DCI, but does not, by itself, establish NVU failure as the primary causal substrate. Vasospasm may remain an important contributor in selected patients, and its pathological significance may vary according to hemorrhage burden, vascular territory, collateral circulation, and the underlying pattern of NVU dysfunction. More broadly, integrating established mechanisms into a common NVU architecture risks becoming overly inclusive if the framework can accommodate virtually any biological abnormality observed after aSAH. The strength of this framework will ultimately depend on whether it can generate specific, testable predictions that distinguish NVU-mediated mechanisms from alternative explanations of DCI.

A further challenge is the proposed heterogeneity of NVU failure. Although the framework suggests that patients may exhibit distinct endothelial-, microvascular-, barrier-, neuroimmune-, or metabolism-predominant patterns, these phenotypes have not yet been defined or validated. The relative contribution and temporal relationship of these compartments may differ substantially between individuals, potentially explaining why single-target interventions have produced inconsistent clinical benefits. This heterogeneity also complicates the clinical identification of DCI, particularly in poor-grade or sedated patients in whom neurological deterioration may be difficult to detect reliably and may not correspond to a single biological mechanism.

Future validation should therefore focus on prospective, longitudinal studies that simultaneously characterize multiple NVU compartments within individual patients from the early brain injury period through the development or resolution of DCI. Multimodal approaches integrating vascular and perfusion imaging with biomarkers of endothelial function, BBB integrity, neuroinflammation, electrophysiological dysfunction, and cerebral metabolism may establish whether early NVU abnormalities consistently precede DCI, identify reproducible biological phenotypes, and determine which components are most predictive of irreversible tissue injury. Critically, these studies should test the framework’s predefined, falsifiable predictions rather than merely demonstrating the coexistence of multiple NVU abnormalities. Therapeutic studies should subsequently determine whether interventions guided by specific NVU phenotypes can improve outcomes beyond conventional pathway-specific treatment, while accounting for the potential toxicity and complexity of combination strategies. Ultimately, the NVU framework should be regarded as a hypothesis-generating framework rather than an established disease model or a replacement for established mechanisms; its validity will depend on whether it can prospectively predict clinical trajectories, identify biologically meaningful patient subgroups, and demonstrate therapeutic implications that cannot be explained by existing paradigms alone.

## 9. Conclusions

DCI after aSAH cannot be fully explained by large-vessel vasospasm alone. The dissociation between angiographic vasospasm, tissue ischemia, infarction, and clinical outcome, together with evidence of early endothelial, microvascular, BBB, neuroimmune, electrophysiological, and metabolic disturbances, supports a broader pathophysiological model.

This perspective proposes, as a hypothesis, progressive NVU failure as a framework that may link these processes. EBI may destabilize neurovascular homeostasis, after which dysfunction across vascular, glial, immune, and neuronal compartments can interact and amplify tissue vulnerability. Within this framework, vasospasm remains an important contributor to DCI in some patients but represents one component of a broader process rather than a sufficient explanation for delayed ischemic injury. Progressive loss of neurovascular and metabolic reserve may ultimately culminate in metabolic failure, tissue injury, and clinical DCI.

This framework remains a hypothesis rather than an established causal sequence. Current evidence does not demonstrate that NVU disturbance occurs in a uniform temporal order or contributes equally across patients, and much of the mechanistic evidence is derived from experimental and observational studies. Longitudinal multimodal studies should determine whether early NVU abnormalities predict subsequent DCI, whether distinct biological trajectories can be identified, and whether targeting multiple NVU compartments can improve outcomes beyond pathway-specific treatment.

An NVU-centered model therefore does not replace vasospasm or other established mechanisms of secondary brain injury. Rather, it offers a hypothesis-generating framework for exploring how these processes may interact to produce DCI and offers a basis for more precise biomarker development, patient stratification, and mechanism-informed therapeutic strategies.

## Figures and Tables

**Table 1:** Integration of Historical DCI Paradigms Within the Neurovascular Unit Framework.

Paradigm	Biological Level	Principal Contribution	Key Limitation	Role	References
Macrovascular Vasospasm	Large arteries	It explains arterial narrowing, regional hypoperfusion, and large-vessel ischemic deficits.	It does not explain infarcts outside spastic areas or outcome failure after vasospasm-targeted trials	It plays a role in upstream vascular output of endothelial NO/ET-1 imbalance that becomes harmful when downstream NVU compensation fails	[58,59]
Early Brain Injury	Tissue	It provides the substrate for later DCI through edema, cell death, endothelial injury, and inflammation	Not a single causal mechanism reported	Initial NVU-wide insult affecting neurons, glia, endothelium, and microvessels before delayed infarction emerges.	[49,59,60]
Neuroinflammation	Cellular & Cytokine Signaling	It explains propagation of injury, leukocyte recruitment, endothelial activation, and thrombogenic signaling	This pathway is interconnected and broad. Therefore, biomarker selection and translation to therapies is difficult.	Immune arm of the NVU that reshapes endothelial permeability, microvascular tone and neuronal survival	[27,49,61]
BBB Dysfunction	Endothelial, pericyte and astrocyte barrier	It explains early permeability change, edema, and exposure of parenchyma to blood-derived mediators	Direct human evidence is still limited and barrier leak does not specify which regions will get affected	Interface whose breakdown exposes the parenchyma to blood-derived mediators and amplifies edema and neuroinflammation	[27,59,61,62]
Cortical Spreading Depolarization	Electrophysiological & Metabolic	it explains cortical infarcts in other areas and abrupt metabolic crises with spreading ischemia.	Not all CSDs are injurious, and timing thresholds remain uncertain	It impairs neurovascular coupling, inducing vasoconstriction instead of vasodilation; activated microglia may both sense and propagate cortical spreading depolarization.	[6,53]
Autoregulatory Failure	Precapillary resistance control	It explains vulnerability to pressure-dependent hypoperfusion and links physiology to spreading depolarization risk	It is hard to measure consistently	Failure of NVU vascular control to stabilize flow against systemic pressure changes	[63]
Oxidative stress	Cellular	It explains hemoglobin-driven injury, endothelial dysfunction, apoptosis, and BBB damage	It usually acts as a cross-cutting amplifier rather than a standalone explanation for spatial heterogeneity	Molecular injury program inside multiple NVU cell types, especially endothelium and parenchyma	[49,61]
Microcirculatory Dysfunction	Microvascular- arterioles, capillaries and venules	It explains vasospasm-independent ischemia, oxygen-delivery failure, and mismatch between angiography and infarction	Mechanistically heterogeneous and harder to monitor directly in patients	Core downstream NVU effector where endothelial cells, pericytes, vSMCs, astrocytes, and neurons lose coordinated coupling.	[49,58]

## Data Availability

No new data were created or analyzed in this study.

## Abbreviations

DCI	Delayed Cerebral Ischemia
NVU	Neurovascular unit
aSAH	Aneurysmal subarachnoid hemorrhage
AUC	Area under the curve
BBB	Blood–brain barrier
CO_2_	Carbon dioxide
CSD	Cortical spreading depolarization
EBI	Early brain injury
GOSE	Glasgow Outcome Scale- Extended
ICP	Intracranial pressure
OR	Odds Ratio
ROS	Reactive Oxygen species
TCD	Transcranial Doppler
NMDA	N-methyl-D-aspartate
GluN2B	Glutamate receptor ionotropic, NMDA 2B
CSF	Cerebrospinal fluid
CT	Computed tomography
ECoG	Electrocorticography
LPR	Lactate-to-pyruvate ratio
NSE	Neuron-specific enolase
GFAP	Glial fibrillary acidic protein
PbtO2	Brain tissue oxygen tension
ET-1	Endothelin-1
NO	Nitric oxide
CPP	Cerebral perfusion pressure
MMP-9	Matrix metalloproteinase-9
mRS	Modified Rankin Scale
vSMC	Vascular smooth muscle cell
